# A profile and review of findings from the Early Markers for Autism study: unique contributions from a population-based case–control study in California

**DOI:** 10.1186/s13229-021-00429-7

**Published:** 2021-03-18

**Authors:** Kristen Lyall, Jennifer L. Ames, Michelle Pearl, Michela Traglia, Lauren A. Weiss, Gayle C. Windham, Martin Kharrazi, Cathleen K. Yoshida, Robert Yolken, Heather E. Volk, Paul Ashwood, Judy Van de Water, Lisa A. Croen

**Affiliations:** 1 A.J. Drexel Autism Institute, Drexel University, Suite 560, 3020 Market St, Philadelphia, PA 19104 USA; 2 Division of Research, Kaiser Permanente Northern California, Oakland, CA USA; 3 Environmental Health Investigations Branch, California Department of Public Health, Richmond, CA USA; 4 University of California, San Francisco, San Francisco, CA USA; 5 School of Medicine, Johns Hopkins University, Baltimore, MD USA; 6 Department of Mental Health, Johns Hopkins University, Baltimore, MD USA; 7 UC Davis MIND Institute, University of California, Davis, Davis, CA USA

**Keywords:** Autism, Risk factors, Immune response, Early Markers for Autism

## Abstract

**Background:**

The Early Markers for Autism (EMA) study is a population-based case–control study designed to learn more about early biologic processes involved in ASD.

**Methods:**

Participants were drawn from Southern California births from 2000 to 2003 with archived prenatal and neonatal screening specimens. Across two phases, children with ASD (*n* = 629) and intellectual disability without ASD (ID, *n* = 230) were ascertained from the California Department of Developmental Services (DDS), with diagnoses confirmed according to DSM-IV-TR criteria based on expert clinical review of abstracted records. General population controls (GP, *n* = 599) were randomly sampled from birth certificate files and matched to ASD cases by sex, birth month and year after excluding individuals with DDS records. EMA has published over 20 papers examining immune markers, endogenous hormones, environmental chemicals, and genetic factors in association with ASD and ID. This review summarizes the results across these studies, as well as the EMA study design and future directions.

**Results:**

EMA enabled several key contributions to the literature, including the examination of biomarker levels in biospecimens prospectively collected during critical windows of neurodevelopment. Key findings from EMA include demonstration of elevated cytokine and chemokine levels in maternal mid-pregnancy serum samples in association with ASD, as well as aberrations in other immune marker levels; suggestions of increased odds of ASD with prenatal exposure to certain endocrine disrupting chemicals, though not in mixture analyses; and demonstration of maternal and fetal genetic influence on prenatal chemical, and maternal and neonatal immune marker and vitamin D levels. We also observed an overall lack of association with ASD and measured maternal and neonatal vitamin D, mercury, and brain-derived neurotrophic factor (BDNF) levels.

**Limitations:**

Covariate and outcome data were limited to information in Vital Statistics and DDS records. As a study based in Southern California, generalizability for certain environmental exposures may be reduced.

**Conclusions:**

Results across EMA studies support the importance of the prenatal and neonatal periods in ASD etiology, and provide evidence for the role of the maternal immune response during pregnancy. Future directions for EMA, and the field of ASD in general, include interrogation of mechanistic pathways and examination of combined effects of exposures.

**Supplementary Information:**

The online version contains supplementary material available at 10.1186/s13229-021-00429-7.

## Background

### Motivation for the EMA study

Autism spectrum disorder (ASD) is a complex neurodevelopmental condition characterized by deficits in social communication and the presence of restricted behaviors or interests [1]. Decades of research have firmly established the importance of genetics in the etiology of ASD [2, 3], although that role is a complex one that is still not fully understood. There is also evidence of substantial involvement of environmental factors in ASD etiology, particularly those acting during the prenatal and early neonatal periods as critical windows of neurodevelopment and susceptibility to environmental influence. Though the pathways to ASD are not fully understood, a growing body of evidence supports immune system dysregulation in risk of ASD [4–7]. Furthermore, fetal exposure to chemicals may also impact neurodevelopment, perhaps through immune pathways [8–10]. However, the mechanisms by which environmental agents interact with the developing immune and nervous systems to cause neurodevelopmental disorders in genetically susceptible individuals are poorly understood. Motivated by these gaps in our knowledge, and the desire to learn more about early biologic processes involved in ASD, the Early Markers for Autism (EMA) study was created in 2004. This review describes the motivation for the EMA study, the methods of this novel population-based case control project, and summarizes major findings published (or accepted) as of October 2020 that serve as unique contributions to the field of ASD epidemiology.

### Historical setting

In the early part of the decade from 2000 to 2010, few studies had directly investigated the role of prenatal immune profiles (e.g., cytokine and chemokine profiles, antibodies to fetal brain proteins, antibodies to specific infectious agents) on neurodevelopmental outcomes in humans [11, 12]. However, findings of immune conditions in the family members of affected individuals, and aberrations in immune markers in both cases and mothers were motivating mechanistic work in this area [13–26]. Also during this time frame, no ASD studies had been conducted measuring biomarker levels of environmental contaminants in samples collected during the prenatal or early neonatal susceptibility periods. Research in related fields suggested developmental concerns for a number of environmental chemicals, including polybrominated diphenyl ethers (PBDEs), polychlorinated biphenyls (PCBs), and certain pesticides [27], but work examining these exposures using prenatal biomarkers for associations with neurodevelopmental outcomes more specifically was truly in its infancy. There was also a gap in knowledge regarding the potential mechanistic pathways of such exposures. When created in 2004, EMA was the first large, population-based, nested case–control study of ASD to utilize archived prenatal and newborn specimens mother-baby pairs. Key features of the EMA study are provided in Table 1.

**Table 1:** Key features of the EMA study

Feature	Benefit for autism epidemiologic study
Population-based	Avoids biases due to selected clinical samples Improves generalizability to source populations
Capitalizes on existing resources (DDS, Vital Statistics, CBP)	Enables relatively large sample size Enables prospective examination of biomarkers through use of archived biospecimens Efficient design- no participant re-contact required
Multiple comparison groups (GP, ID)^a^	Enables consideration of specificity of associations to ASD (ASD vs GP) vs broader developmental delays (ID vs GP and ASD vs ID comparisons)
Inclusion of biospecimens collected during critical developmental periods prior to diagnosis	Allows for measurement of exposure levels during mid-pregnancy or in newborn period, rather than estimation via reported or recalled information Availability of a wide range of biomarkers (see Table 3)
Case confirmation via clinician review of records	Enhances confidence in validity of diagnostic categories under study Reduces potential for outcome misclassification
Rich dataset of multiple exposures/biomarkers	Allows for examination of combined effects Ability to examine mechanisms through pathway analyses considering intermediate biomarkers (e.g., thyroid hormones, immune markers as intermediates between EDCs and outcomes)
Interdisciplinary team	Supports cross-cutting science Early stage investigator engagement and mentorship supported^b^

*ASD* autism spectrum disorder, *CBP* California Biobank Program, *DDS* Department of Developmental Services, *EDCs* endocrine-disrupting chemicals, *GP* general population controls, *ID* intellectual disability

^a^The EMA study also included a small number of siblings (67 siblings of those with ASD and 65 of those with ID) to enable additional within-family sibling comparisons; however, these have not been utilized in studies summarized here to date

^b^Over 70% of EMA publications to date have been led by early stage investigators, often stimulating career development

### Goals and opportunities

The EMA Study sought to advance our knowledge of the effects of immunologic, genetic, and environmental factors, individually and in combination, on ASD etiology, utilizing biomarkers of exposures and examining immune and endocrine pathways. With a large, essentially population-based sample of children with confirmed ASD, children with other developmental disabilities, as well as unaffected children, we had an unprecedented opportunity to explore the interplay between genetic susceptibility and markers of immune function and environmental exposures that contribute to ASD risk. Furthermore, because banked biospecimens were being used, the design enabled us to prospectively investigate measured levels of these factors during critical periods of fetal brain development in a novel way. We focused on exposures and markers that were known or suspected to play a role in early brain development. These included antibodies to viral infections, cytokines, chemokines, antibodies to fetal brain proteins, neurotrophins, hormones, metals, and endocrine disrupting chemicals (EDCs) that could be measured in these archived maternal blood (sera and cell pellets) collected in mid-pregnancy and neonatal blood spots collected in the first few days of life. We anticipated that findings from this study would define areas for further investigations of physiologic mechanism, provide a better understanding of the underlying biology involved in ASD etiology, and provide normative data on unaffected controls that may be useful as benchmarks for future studies utilizing prenatal and neonatal specimens.

## Methods

### Study population

**Fig. 1:**
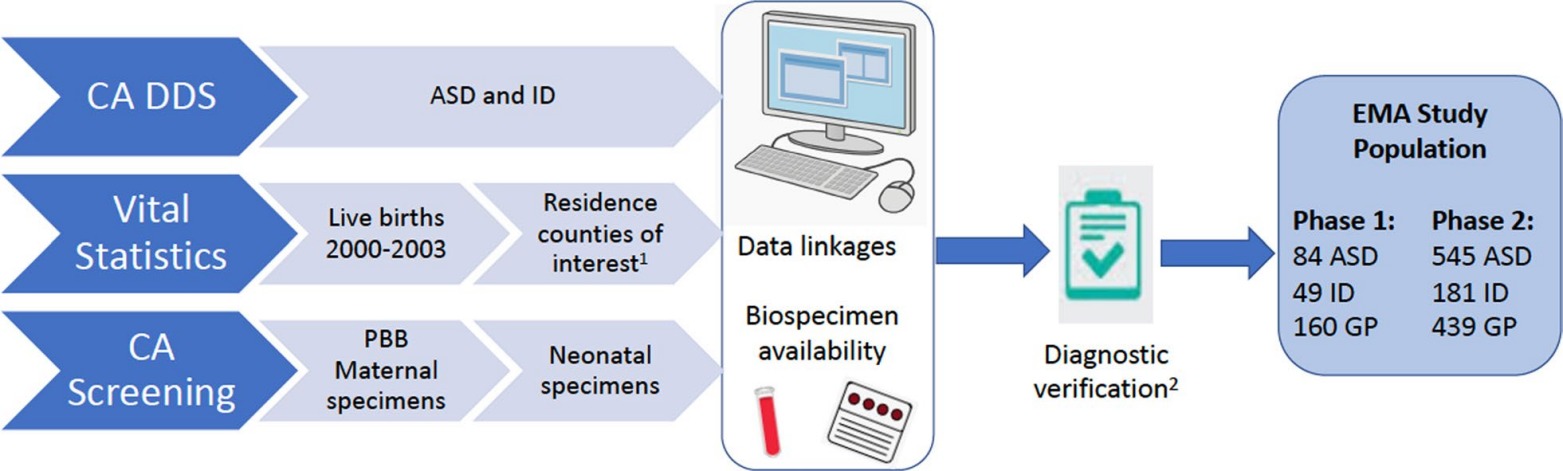
EMA study sampling and overall study design framework. Final analytic samples listed for phases represent the total study populations; numbers in specific genetic and biomarker analyses varied (see Table 4). ^1^Residence counties included: Orange (phase 1 and 2), Imperial (phase 2), and San Diego (phase 2). ^2^Controls were originally oversampled 2:1 to cases. CA DDS, California Department of Developmental Services; ASD, autism spectrum disorder; ID, intellectual disability; GP, general population controls; PBB, Project Baby’s Breath

The EMA study framework is depicted in Fig. 1. The EMA study population was drawn from a 2000–2003 cohort of births in Southern California in which mother’s prenatal screening specimens were archived as part of Project Baby’s Breath (M Kharrazi and GN DeLorenze, Co-PIs) [28]. Women who participated in the prenatal expanded alphafetoprotein screening program (XAFP), [29] offered to all pregnant women in California in the second trimester of pregnancy, during 1999–2002 in Orange, Imperial, and San Diego Counties were eligible for participation in Project Baby’s Breath; in addition to this participation in screening, for inclusion in EMA, women who had a live born infant for whom a California birth certificate match could be found (approximately 90% of screening participants), and for whom both a maternal prenatal screening blood specimen and a newborn screening blood specimen were available in the State archives, were eligible. EMA was conducted in two phases; the first phase was drawn from women who were pregnant in Orange County, California, and who delivered a live born infant from July 2000 to September 2001. The second phase was drawn from women who were pregnant in Orange, San Diego, or Imperial Counties in Southern California and delivered a live born infant from January 2000 to June 2003, after excluding those in the first phase, thereby serving to expand the sample size, geographic region, and birth year range of the first phase. This geographic region included urban, suburban and rural areas in the southern part of California closest to the Mexico border with diverse geographies and race/ethnicities. Study activities were approved by the Institutional Review Board at Kaiser Permanente and the State Committee for the Protection of Human Subjects. Informed consent was not required for the EMA study, given that consent forms for screening programs were distributed at the time of collection, which stipulated that specimens and results from prenatal and neonatal testing could be used for research purposes given IRB approval.

*Representativeness:* Approximately 70% of pregnant women participated in the XAFP in CA during this time period [29, 30]. Screened women were more likely to be under age 35, but were similar to the total population of women delivering in study counties for these birth years with respect to maternal education, race/ethnicity, parity, and health insurance status (Additional file 1: Table S1). Basic characteristics of the EMA study population, by phase and study group, are provided in Table 2.

**Table 2:** Demographic characteristics of the EMA study population by study phase and diagnostic group

	Phase 1			Phase 2		
	ASD *N* = 84	ID *N* = 49	GP *N* = 160	ASD *N* = 545	ID *N* = 181	GP *N* = 439
Maternal age, mean (std)	30.9 (5.2)	28.3 (5.2)	28.2 (5.5)	29.9 (5.6)	27.2 (6.3)	28.7 (5.4)
Paternal age, mean (std)	34.0 (6.3)	33.0 (7.9)	31.0 (6.4)	35.7 (14.6)	36.9 (21.2)	34.0 (14.2)
Child gestational age in days, mean (std)	272.1 (18.5)	266.0 (27.9)	271.1 (14.2)	274.3 (27.1)	268.2 (30.2)	279.0 (36.1)
Child sex (%)						
Male	87%	59%	88%	82%	57%	83%
Female	13%	41%	12%	18%	43%	17%
Multiparous (%)	50%	67%	58%	55%	66%	62%
Maternal birth place (%)						
US	54%	33%	45%	50%	44%	48%
Mexico	11%	45%	36%	24%	46%	31%
Other	36%	22%	19%	26%	10%	21%
Maternal race/ethnicity (%)						
Non-Hispanic White	44%	18%	34%	35%	18%	33%
Asian	23%	18%	18%	15%	5%	11%
Black, Pacific Islander, Other	7%	6%	3%	9%	7%	8%
Hispanic	24%	57%	46%	40%	70%	47%
Missing	2%	0%	0%	1%	0.6%	0.7%
Maternal education (%)						
Less than high school	8%	47%	31%	18%	42%	24%
High school	18%	18%	25%	22%	28%	27%
Some college/college degree	48%	27%	29%	41%	23%	34%
Post-graduate	25%	8%	16%	18%	6%	13%
Missing	1%	0%	0%	1.5%	1%	1%
Intellectual disability (%)^a^						
Yes	40%	100%	0%	52%	100%	0%
No	36%			41%		
Unknown	24%			7%		

*ASD* autism spectrum disorder, *ID* intellectual disability group without ASD as defined in text, *GP* general population controls

^a^Defined according to scores < 70 on cognitive tests in the DDS record

*Group ascertainment*: Three groups of children born to women in the cohort were identified: children with ASD, children with intellectual disability without ASD (ID; formerly classified as mental retardation or developmental delay in some EMA publications), and general population (GP) controls. Individuals with ID of known etiology (e.g., Down’s syndrome) were excluded. Further definition of these groups is provided below in diagnostic verification. Children with ASD or ID were ascertained from the California Department of Developmental Services (DDS), which operates a system of 21 Regional Centers (RC) that coordinate services for persons with ASD and other developmental disabilities. Services are provided without regard to citizenship or financial status; thus, the system is widely utilized across socio-economic levels and racial/ethnic groups. DDS maintains a centralized electronic database of diagnostic and identifying information on all clients, derived from information provided by the RCs on a standardized, statewide reporting form (Client Development Evaluation Report). Referrals to the RCs come from pediatricians and other clinical providers, the education system, friends, and family members. DDS and the RCs are mandated to provide services to individuals with autistic disorder and children with other Pervasive Developmental Disorders (PDDs) who have ID (IQ < 70) or are substantially impaired. Children with milder forms of ASD were less likely to be captured in these records. GP controls were randomly sampled from the birth certificate files, frequency matched to ASD cases by sex, birth month and birth year, after excluding all past or current DDS/RC clients. Controls were originally sampled to ensure a 2:1 control-ASD case ratio after retrieval of maternal and neonatal specimens. Among those with live births in the study counties and birth years, ~ 50% across all study groups linked to stored specimens. (This proportion is lower than the overall participation rate in the XAFP due to variability in participation rates over time and region, and ability to link to stored specimens). At the time of case and control ascertainment, children were approximately 3–4 years old for phase 1 and 4.5–9 years for phase 2.

### Diagnostic verification and case classifications

Following a protocol initially developed by the CDC, trained medical record abstractors reviewed and abstracted detailed diagnostic and clinical data from RC records for all children identified as ASD and ID for whom archived blood specimens were found. Final case status was determined by expert clinical review of the abstracted information, according to DSM-IV-TR criteria. The overall EMA participant selection process is shown in Fig. 1. Most of those ascertained as ASD cases were confirmed (72% for phase 1 and 98% for phase 2), while 22% (phase 1) and 38% (phase 2) of those originally identified as ID were reclassified as ASD cases according to expert clinician review of records. After this expert review, final analytic samples consisted of 84 children with ASD and 49 with ID for phase 1 and 545 children with ASD and 181 children with ID for phase 2. Certain analyses across phases also considered ASD subtypes, including early onset or regressive (phase 1), and presence or absence of co-occurring ID (phase 1 and 2). Determination of ID (and co-occurring ID within the presence of ASD) was based on composite scores < 70 on all standardized cognitive and functional tests recorded in RC records (with no ID defined according to all or some scores > 70, and unknown ID defined according to the absence of standardized scores in chart).

### Covariate information

Data were obtained through California Vital Statistics, the GDSP, and the DDS. Parental demographics, including parental age, dates of birth, education level, race, ethnicity, country of origin, and insurance status at delivery were obtained through birth certificates. Additional maternal information, including weight, height, and pregnancy-related characteristics were obtained at time of prenatal sample collection. Infant death and neonatal information, including birthweight and gestational age, was obtained through Vital Statistics. While covariates included in adjusted models varied across studies, study matching factors, maternal age, weight at prenatal specimen collection, and race/ethnicity were typically adjusted for in analyses; additional adjustment factors were considered as appropriate for analyses (see also Table 4).

### Retrieval of biospecimens

Maternal mid-pregnancy (range 15–20 weeks gestation) blood specimens were obtained from Project Baby’s Breath and dried newborn bloodspots were obtained from the CA Genetic Disease Screening program (GDSP) of the California Biobank Program (https://www.cdph.ca.gov/Programs/CFH/DGDS/Pages/cbp/default.aspx). All biospecimens were retrieved from -20 C freezer archives, barcoded with a unique study identification number, and shipped to the participating laboratories. For all subjects, one whole dried bloodspot, 1–2 ml (mean 1.8 ml, SD 0.3 ml) of maternal serum, and 1-2 ml of maternal blood cell pellet were retrieved.

### Laboratory and exposure analyses

Biomarkers measured in specimens from EMA participants are shown in Table 3 (see also Additional file 1: Appendix). Laboratory procedures for analytes have been previously published. We refer the reader to individual papers for more detailed information on methods for a given biomarker; those examined in multiple EMA studies are briefly described here. Cytokines and chemokines were measured in maternal serum and newborn samples using commercially available multiplex bead-based kits, with assays carried out following manufacturer protocols [31, 32]; TSH was measured in regional laboratories as part of routine screening in maternal serum using an I-labeled immunoradio-metric assay and newborn bloodspots using solid-phase, time-resolved sandwich fluoroimmunoassay [33, 34]. Vitamin D was measured as total 25-hydroxyvitamin D (sum of 25(OH)D2 and 25(OH)D3, hereinafter referred to as 25(OH)D) by a sensitive isotope dilution liquid chromatography-tandem mass spectrometry method (LC/MS/MS) in both maternal serum and dried newborn blood spots (converted to serum equivalents) [35–37]. Endocrine disrupting chemicals (PCBs, OCPs, PBDEs, and PFASs) were measured in maternal serum samples using gas chromatography isotope dilution high resolution mass spectrometry, [38–40].

**Table 3:** Biomarkers and exposures measured in the EMA Study, by study phase and time point

Category	Biomarker/exposure	Phase 1		Phase 2	
		Maternal	Neonatal	Maternal	Neonatal
Immune-related factors	Cytokines (IL-2, IL-4, IL-5, IL-6, IL-10, IL-13, IL-1B, INF-γ, TNF-a)^a^	X	X	X	X
	Chemokines (IL-8, IL-12, IP-10, MCP-1, MIP-1α, MIP-1β, RANTES, GM-CSF, Eotaxin)^a^	X	X	X	X
	C-reactive protein (CRP)	X		X	
	Antibodies to Infectious Agents (CMV-IgG, EBV-IgG, HHV-6-IgG, HSV-1, HSV-2, Influenza A, Influenza B, VZV, Toxo-IgG, Toxo-IgA, Toxo-IgM)	X (Toxo only)	X		
	Autoantibodies to fetal brain protein	X		X	
	Immunoglobulins (IgG, IgM, IgA, IgE)	X	X (no IgE)		
Additional endogenous factors	Hormones (TSH, SHBG, bioactive androgen)	X	X (TSH only)		X (TSH only)
	Brain-derived neurotrophic factor (BDNF)	X	X		
	25(OH)D			X	X
Environmental chemicals	EDCs including: PBDEs (6 of 10 congeners sufficiently detected^b^: BB153, BDEs-28, -47, -99, -100, and -15) PCBs (11 of 37 congeners sufficiently detected: 28, 99, 118, 138/158, 153, 170, 180, 187, 194, 196/203, 199) OCPs (2 of 11 pesticides sufficiently detected^b^: *trans*-nonachlor, p,p’-DDE) PFAS (8 measured: Et-PFOSA-AcOH, Me-PFOSA-AcOH, PFHxS, PFNA, PFOA, PFOS sufficiently detected^b^; PFDeA and PFOSA examined as binary detect/non-detect variables)			X	
	Mercury	X	X		
	Prenatal air pollution exposure: PM_2.5_, PM_10_, O_3_, NO_2_			X	
Genetic factors	Candidate genes (HERV-K, BDNF, CD48, ApoE, Ltabtnf1, Apobec3g, OAS1, IL-1α (889), IL-1β (511), IL-6 (174), IFN-γ, IL-10 (1082, 592, 819)	X	X		
	GWAS			X	X
	CNVs			X	

*GWAS* genome wide association study, *TSH* thyroid stimulating hormone, *BDNF* brain derived neurotrophic factor, *25(OH)D* 25-hydroxyvitamin D (vitamin D), *EDCs* endocrine disrupting chemicals, *PBDEs* polybrominated diphenyl ethers, *PCBs* polychlorinated biphenyl ethers, *OCP* organochlorine pesticide, *PFAS* perfluoroalkyl substances. Maternal biomarkers were measured in mid-pregnancy (15–19 weeks gestation) serum samples; neonatal biomarkers were measured from newborn blood spots

**Fig. 2:**
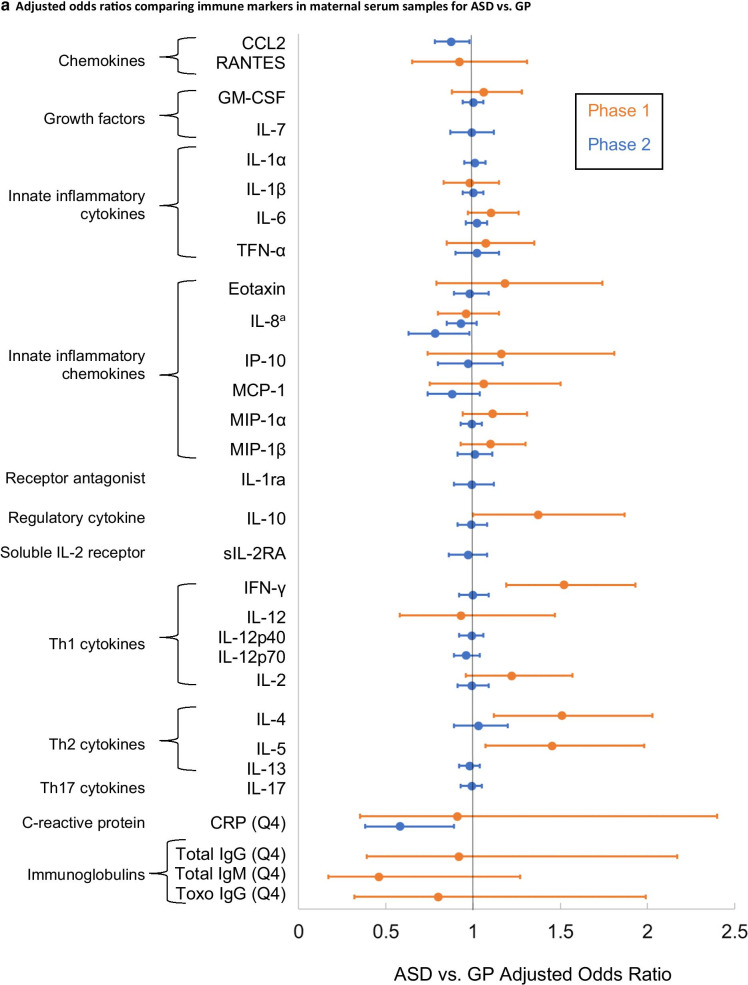

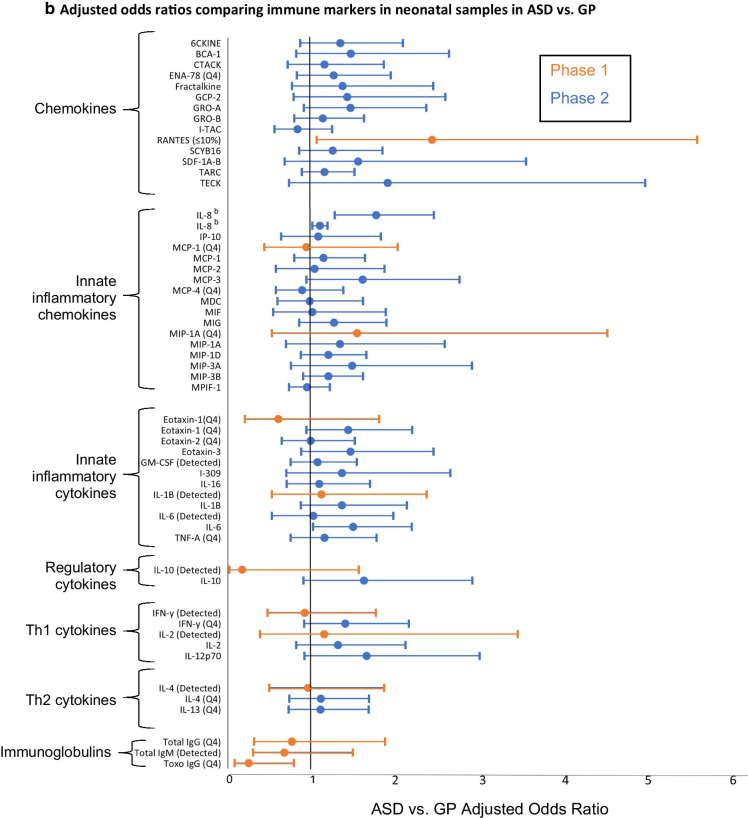
Forest plot demonstrating adjusted ORs (points) and 95% confidence intervals (lines) of the association between individual immune markers (listed along the Y axis) measured in (**a**) maternal mid-pregnancy serum samples and (**b**) in newborn bloodspots and ASD. ORs represent 1-SD change in immune marker, or as indicated according to the highest quartile (Q4) versus the lowest. **a** Most estimates are adjusted for gestational age at the time of draw, maternal weight, age, race, ethnicity and country of origin. References: Goines et al. [31], Zerbo et al. [49], Grether et al. [54], Jones et al. [55], Traglia et al. [43]. 6CKINE, chemokine (C–C motif) ligand 21 (CCL21); BCA-1, B cell-attracting chemokine 1; BDNF, brain-derived neurotrophic factor; CRP, C-reactive protein; CTACK, cutaneous T cell–attracting chemokine; GCP-2, granulocyte chemotactic protein 2; IFN, interferon; IL, interleukin; MCP-4, monocyte chemotactic protein; MIP-1a, macrophage inflammatory protein 1 alpha; Q4, OR compares highest quartile to lowest quartile; TNF, tumor necrosis factor. ^a^IL-8 was examined in phase 2 data in Jones et al. [55] and replicated in a genetic subsample of phase 2 in Traglia et al. [43]. **b** Most estimates are adjusted for birth type (C-section vs. vaginal), child’s sex, gestational age at birth, birth weight, postnatal age at bloodspot collection, maternal and paternal age, maternal and paternal education, birth season, maternal birth place, child’s birth year, maternal and paternal race, and Bio-Plex plate number. References: Zerbo et al. [32], Grether et al. [54], Traglia et al. [43], Heuer et al. [56]. ≤ 10%, OR compares ≤ 10% marker quantile to > 10% level; 6CKINE, chemokine (C–C motif) ligand 21 (CCL21); BCA-1, B cell-attracting chemokine 1; CTACK, cutaneous T cell–attracting chemokine; Detected, OR compares value above the minimum level of detection to values below; GCP-2, granulocyte chemotactic protein 2; Gro-α, growth-regulated oncogene alpha; IFN, interferon; IL, interleukin; MCP, monocyte chemotactic protein; MIP-1a, macrophage inflammatory protein 1 alpha; MIG, monkine induced by gamma interferon; Q4, OR compares highest quartile to lowest quartile; TARC, thymus and activation regulated chemokine; TECK, thymus-expressed chemokine; TNF, tumor necrosis factor. ^b^IL-8 was examined in phase 2 data in Heuer et al. [56] and in a genetic subsample of phase 2 in Traglia et al. [43]

^a^A larger set of cytokines and chemokines were measured in phase 2 than in phase 1; see Fig. 2a, b and Additional file 1: Appendix for a complete list

^b^Defined as detected above the LOD in at least 60% of the study population

Air pollution in EMA was assigned as averaged monthly exposure for the month prior to maternal serum collection based on the Environmental Protection Agency’s Air Quality System data (http://www.epa.gov/ttn/airs/airsaqs) for nitrogen dioxide (NO_2_), ozone, and particulate matter less than 10 and 2.5 microns in diameter (PM_10_, PM_2.5_) using the maternal residential address reported during the prenatal screening visit.

DNA was extracted from a subset of 790 maternal cell pellets and 764 neonatal bloodspots and 675,000 SNPs were genotyped across the genome using standard protocols and quality assessment, as previously described [41]. The genotyped samples included 366 ASD mother–child pairs and 369 control pairs [42, 43]. The ID cases and ASD cases identified through reclassification of those originally enrolled as ID were not included in the genome-wide genotyping due to budget limitations, but were used for analyzing specific candidate genetic factors [44].

### Genetic approach

Given that EMA is comprised of mother–child pairs of cases and unrelated controls, we were able to take advantage of some features of family-based analyses. We considered models where the maternal genetic make-up could act as a determinant of the fetal environment, impacting the early risk for ASD. We tested for maternal main effects influencing risk for ASD, as well as maternal-child genetic combinations or interactions that might impact risk [41]. With genotyping data, we also estimated copy number variants (CNVs) and tested for maternal effects that might be mediated through CNVs [45]. Combining EMA genetic and biomarker data we conducted G × E analyses. Previous studies using genome-wide association study (GWAS) data to identify G × E interactions encountered statistical power issues compared with the detection of main effects [46, 47]. We hypothesized that the most likely genetic candidates for G × E would be the variants associated with the E biomarker itself. Our strategy was thus to perform a quantitative trait locus (QTL) analysis to identify candidate genetic factors that contribute to the inter-individual variation of the E biomarker and then test for interaction with case–control status among those top candidates [42, 43]. Given the variety of genetic ancestries in the EMA study population, methods appropriate for mixed-ancestry data were used to avoid ancestry-related false positives, such as adjustment for genome-wide principal components [48]. In addition, because mothers and their affected children are genetically correlated with each other, we performed separate GWAS to analyze distinct maternal and neonatal effects. When we observed both maternal and fetal genetic contribution for the same biomarker, we identified the offspring alleles not inherited from the maternal lineage (fetal-specific alleles) and the maternal alleles not transmitted to the offspring (maternal-specific alleles) in order to distinguish whether both maternal and fetal genomes independently affected a specific biomarker [43].

We also applied the principles of Mendelian randomization to assess the possible causal relationship of a risk trait with ASD outcome. If a trait (i.e. immune biomarker or vitamin D) is causally linked to ASD, the genetic variants influencing the trait should also influence ASD risk. We thus tested the association between the mapped genetic determinants of the trait in the EMA dataset and ASD outcome [42, 43, 49].

## Major EMA findings

A summary of EMA findings for associations with ASD from publications through October 2020 is provided in Table 4. Brief descriptions of key findings within each of the major target areas of interest are further provided below.

**Table 4:** Summary of EMA studies published as of October 2020

Category	References	EMA phase and n	Focus^a^	Primary finding(s)
Immune-related factors	Croen et al. [50]	1 84 ASD, 49 ID, 160 GP	Antibody reactivity in maternal samples	Maternal mid-gestation antibody reactivity to human fetal brain protein differed by study group and by autism onset type, though most differences did not reach statistical significance. Reactivity to a band at 39 kDa was more common in mothers of children with autism (7%) compared with mothers of ID and GP controls (0% and 2%, *p* = 0.09 and 0.07, respectively). Reactivity to bands at 39 kDa and 73 kDa was found only in mothers of children with early onset autism (*n* = 3)
	Goines et al. [31]	1 84 ASD, 49 ID, 159 GP	Cytokine and chemokines in maternal samples	Differing cytokine profiles observed in ASD and ID groups relative to GP controls: Elevated levels of IFN-γ, IL-4, IL-5 in midgestation maternal serum were significantly associated with a 50% increased risk of ASD (AOR = 1.51, 95% CI 1.12, 2.03, for IL-4 and similar for IL-5) while elevated IL-2, IL-4 and IL-6, and GM-CSF and chemokine MIP-1α, were significantly associated with an increased risk of ID without autism (AOR = 2.18, 95% CI 1.24, 3.85 for IL-4 and AORs ranging from 1.22 to 1.72 for others). In both ASD and ID IL-10 was elevated relative to GP controls
	Grether et al. [54]	1 84 ASD, 49 ID, 159 GP	Immunoglobulin levels in maternal and newborn samples	Inverse associations between immunoglobulin levels and ASD: Higher Toxo IgG associated with lower risk of ASD in both maternal and newborn specimens (AOR for newborn = 0.25, 95% CI 0.08, 0.78, similar for maternal though attenuated). Overall lower immunoglobulin levels associated with higher ASD risk, but most did not reach statistical significance. No associations seen for ID
	Zerbo et al. [32]	1 84 ASD, 49 ID, 159 GP	Cytokine and chemokines in newborn samples	Cytokines were not detected in most newborn samples, regardless of case or control status. However, certain chemokines were associated with ASD and ID: MCP-1 was elevated and RANTES was decreased in ASD cases compared to GP controls; MIP-1α and RANTES were both decreased in children with ID compared to GP controls
	Zerbo et al. [49]	1, 2 500 ASD, 235 ID, 580 GP	CRP in maternal samples	Maternal CRP levels were lower in mothers of ASD cases compared with GP controls. Maternal CRP levels in the upper quartile were associated with a decreased risk of ASD (AOR = 0.58, 95% CI 0.38, 0.89). No difference was found between maternal CRP levels of ID and GP controls
	Jones et al. [55]	2 415 ASD, 188 ID, 428 GP	Cytokines and chemokines in maternal samples	Mothers of children with ASD + ID had significantly elevated levels of a number of cytokines and chemokines, including GM-CSF, IFN-γ, IL-1α, and IL-6, compared to mothers of children with either ASD without ID, those with ID, or GP controls. Mothers of children with either ASD-without ID or with ID had significantly lower levels of the chemokines IL-8 and MCP-1 compared to mothers of GP controls
	Heuer et al. [56]	2 370 ASD, 140 ID, 378 GP	Cytokines and chemokines in newborn samples	Children with ASD had significantly increased neonatal levels of interleukin-6 (IL-6) and IL-8 compared with GP controls. In particular, higher IL-8 was associated with ASD with early onset. Children with ASD also had significantly higher levels of eotaxin-1, interferon-γ, and IL-12p70 compared to children with ID. No significant differences were noted between the ID and GP groups
Additional endogenous factors	Croen et al. [50]	1 84 ASD, 49 ID, 159 GP	BDNF in maternal and newborn samples	Prenatal and neonatal BDNF levels were not significantly associated with ASD or ID
	Yau et al. [34]	1 84 ASD, 49 ID, 159 GP	Thyroid stimulating hormone levels in maternal and newborn samples	Inverse associations between ASD and TSH levels in maternal serum samples (ASD vs. GP: AOR 0.33 95% CI 0.12–0.91, Early Onset ASD vs. GP: 0.31 95% CI 0.10–0.98). Results for thyroid levels in newborn blood samples were similar though not significant (ASD vs. GP: AOR = 0.61, 95% CI 0.18–2.04). Among children with ID vs. GP, relationships between maternal TSH (AOR = 0.9, 95% CI 0.02–0.42) and neonatal TSH (AOR = 0.36, 95% CI 0.08, 1.61) were similar to ASD vs. GP findings
	Ames et al. [33]	2 518 ASD, 145 ID, 399 GP	Thyroid stimulating hormone in newborn samples	No association between neonatal TSH levels and ASD vs. GP nor ID vs GP. Among ASD sub-phenotypes, a suggestive inverse trend was observed between ASD with regression and TSH, though the association was only statistically significant in the highest TSH quartile (AOR: 0.50, 95% CI: 0.26–0.98)
	Windham et al. [37]	2 534 ASD, 181 ID, 421 GP	Neonatal vitamin D levels	No association between newborn 25OHD levels and ASD nor ID, but potential differences by sex and ethnicity observed. In non-Hispanic whites, as well as in male children, a protective effect of maternal 25OHD was observed (AOR for non-Hispanic whites = 0.82, 95%CI 0.69–0.98 per 25 nmol/L; similar in males), and suggestion of non-linear effects (inverted j-shaped) for the latter. In females, an increased risk with higher 25OHD was observed
	Windham et al. [71]	2 534 ASD, 181 ID, 421 GP	Maternal vitamin D levels	No overall associations between maternal 25OHD levels and ASD nor ID. Non-linear, inverted j-shape pattern between maternal 25OHD and ASD (*p* = 0.009 for linearity), and suggestion of interactions with sex and ethnicity (AOR AOR = 0.82, 95%CI 0.69–0.9 for non-Hispanic Whites, similar for males), were observed, as for neonatal vitamin D
Environmental chemicals	Yau et al. [83]	1 84 ASD, 49 ID, 159 GP	Mercury levels in maternal and newborn samples	No statistically significant associations found with either maternal or newborn levels, though there were non-significant elevations in odds of ASD with higher levels of mercury, and odds of ASD relative to ID were higher for higher quartiles of mercury
	Lyall et al. [3]	2 545 ASD, 181 ID, 418 GP	Organochlorine chemicals (OC pesticides and PCBs) in maternal samples	General pattern of increased odds of ASD with higher maternal PCB levels, particularly for certain PCB congeners (138/158, 153, 170, and 180; AOR for Q4 v Q1 = 1.82, 95% CI 1.10, 3.02 for PCB153, similar though attenuated for most others). No significant associations were seen for ASD and the two pesticides examined (p,p’-DDE and trans-nonachlor). Results were similar for ID, though the 3rd quartile of p,p’-DDE showed a significantly increased risk of ID relative to the first (1.89, 95% CI 1.03, 3.44), though no trend was suggested
	Lyall et al. [39]	2 545 ASD, 181 ID, 418 GP	PBDEs in maternal samples	General pattern of reduced odds of ASD with higher maternal brominated flame retardant levels; significant associations were observed for certain congeners (BDE-153, as well as the sum across BFR congeners, AOR 0.54, 95% CI 0.36, 0.80). In analyses of ASD stratified by sex, estimates for all BFR congeners except BB153 suggested positive associations for female children, but inverse associations for male children. Results were similar for ID
	Lyall et al. [38]	2 553 ASD, 189 ID, 433 GP	PFASs in maternal samples	Associations with maternal PFASs were null to inverse; those with the highest levels of PFOA had reduced odds of ASD (Q4 vs. Q1 AOR = 0.62; 95% CI: 0.41, 0.93). Associations were similar for ID
	Hamra et al. [79]	2 491 ASD, 155 ID, 373 GP	EDCs listed above	Applying a Bayesian mixture approach, no significant associations with EDCs and ASD nor ID were observed, even for chemicals identified in prior analyses of individual chemicals. AORs were all close to the null
	Volk et al. [82]	2 379 ASD, 164 ID, 414 GP	Air pollution and cytokines	Observed several relationships between air pollution exposure (PM_2.5_, PM_10_, O_3_, NO_2_,) during the prior month and maternal serum cytokine and chemokine levels measured mid-gestation. However, no direct relationships of air pollutants and neurodevelopmental outcomes (ASD+/I ID, ID only), nor was there evidence of mediation by maternal mid-pregnancy immune response
Genetic factors^b^	Tsang et al. [41]	2 390 ASD, 400 GP for maternal and 385 ASD, 379 GP for neonatal	Transgenerational genetic effects	Associations with SNPs in or near several genes previously implicated in autism (including NLGN4X, RAPGEF4, RORA, FAM135B and CNTNAP2) were identified in mothers of ASD cases, though no results reached genome-wide significance. Transgenerational epistasis analysis found several suggestive associations (at the *p* < 10^−4^ level)
	Desachy et al. [45]	2 349 ASD and 351 GP	Maternal CNVs	Higher autosomal burden of large, rare CNVs in females in ASD families (though not unique to ASD). Case mothers had more CNVs (both deletions and duplications) than control mothers, although this difference was not significant for duplications
	Traglia et al. [42]	2 390 ASD, 400 GP for maternal and 385 ASD, 379 GP for neonatal	Genes and EDC levels	Maternal circulating levels of BB-153, BDE-47, -100, -153 and their sum were significantly controlled by common genetic factors, and maternal genome-wide significant associations were observed between p,p’-DDE and PBDE levels and a SNP (rs7259965 which maps within the CYP2B6 locus). In addition, mid-gestational levels of BB-153, BDE-47, -99, -100, -153 and the sum of PBDE congeners were more strongly regulated by fetal genetic factors than maternal genetics
	Traglia et al. [43]	2 390 ASD, 400 GP for maternal and 385 ASD, 379 GP for neonatal	Genes and immune markers	Results supported a large contribution of genetic variation in regulating cytokine/chemokine status during pregnancy and at birth. Neonatal levels of immune molecules were not correlated with mid-gestational maternal levels. 17 specific maternal and fetal loci were identified as contributing to cytokine and chemokine status at the two different time points. Distinct maternal loci in novel regions were independently associated with cytokine/chemokine levels in newborns, and fetal loci in novel regions were independently associated with maternal cytokine/chemokine levels. Increased IL-8 was associated with ASD status only in the presence of a specific maternal SNP genotype
	Traglia et al. [44]	2 507 ASD, 179 ID, 400 GP for maternal and 385 ASD, 379 GP for neonatal	Genes and vitamin D	Evidence for a genome-wide significant missense variant located in the GC gene (previously identified as part of the vitamin D pathway), influencing neonatal vitamin D levels. Novel loci from maternal and fetal genetics emerged at suggestive levels near genes important for immune function. For ID, an association between decreased neonatal vitamin D levels and mothers with a specific genotype was observed

CNV, copy number variant; CRP, C-reactive protein; BDNF, brain-derived neurotrophic factor; GM-CSF, granulocyte–macrophage colony-stimulating factor; MCP-1, monocyte chemotactic protein-1; RANTES, Regulated upon Activation Normal T-Cell Expressed and Secreted; OC, organochlorine; PCB, polychlorinated biphenyl; PBDE, polybrominated diphenyl ether; PFAS, per- and polyfluorolalkyl substances; EDC, endocrine disrupting chemicals (includes OC pesticides, PCBs, PBDEs, and PFASs); AOR, adjusted OR. Covariates included in adjusted models varied across studies but typically included study matching factors, maternal age, weight at prenatal specimen collection, and race/ethnicity; additional study-specific adjustment factors may have included maternal education, PCs for genetic ancestry, maternal country of birth, and gestational age for certain neonatal associations

^a^Analytes measured in maternal samples collected in SST serum tubes at an average of 15–19 weeks gestation; those measured in newborn samples from dried newborn bloodspots collected on average 24–48 h after birth.

^b^GWAS data were not obtained for maternal-child pairs in the ID-only group overall. For Traglia et al. [44], a subset of candidate markers was genotyped in mother–child pairs in the ID-only group

### Immune markers

EMA has contributed 7 studies focused on the role of immune markers in ASD, many of these among the first to examine such markers in prospectively collected prenatal and neonatal biospecimens. Summaries of adjusted associations between ASD and immune markers in maternal and neonatal specimens are provided in Fig. 2a, b, respectively. Immune markers measured in EMA, by phase, are shown in these figures; those measured in phase 2 (which expanded phase 1 measures) are also listed in the Appendix. Results generally supported associations in the same direction across Phases (with the exception of neonatal eotaxin and IL-10), although the samller sample size in Phase 1 yielded less precise estimates. Across these studies, there was a general pattern of moderate increases in ASD risk with inflammatory cytokines; additional patterns are highlighted further below.

One of EMA’s first publications demonstrated a relationship between maternal antibodies to fetal brain proteins measured in mid-pregnancy samples and ASD [50], a result consistent with earlier work suggesting similar findings in maternal samples collected after the birth of the child, as well as in animal models [51–53]. Another investigation in phase 1 suggested lower levels of the immunoglobulin IgG to *Toxoplasma gondii* in both maternal and neonatal samples were associated with increased risk of ASD, but no differences were noted in the ID group [54]. In addition, work conducted in the phase 2 sample suggested lower levels of C-reactive protein (CRP, acute phase protein produced in response to inflammation) in maternal samples of ASD cases, or significantly reduced risk of ASD with higher levels of CRP [49]. Together, these results could suggest an impaired response to immune activation in mothers of ASD cases.

Examination of cytokine profiles from phase 1 [31] demonstrated differences potentially suggestive of an asthma/allergy phenotype in mothers of ASD children, with elevated concentrations of IFN-γ, IL-4 and IL-5. A more inflammatory phenotype was found in mothers of ID children, characterized by elevated concentrations of IL-2, IL-4, and IL-6. This work was expanded to examine a larger panel of cytokines and chemokines levels in a greater number of maternal samples from phase 2, which demonstrated elevated maternal levels of several proinflammatory cytokines and chemokines in association with ASD with ID [55]. This differentiated the ASD with ID group from all other groups including ASD without co-occurring ID, ID without ASD, and GP controls. When neonatal samples were similarly examined, higher levels of inflammatory cytokines were found in ASD cases relative to GP controls, and other inflammatory T cell cytokines and certain chemokines were elevated in ASD relative to ID [56].

Results across these studies of immune markers therefore suggest immune dysregulation, in some cases consistent with pro-inflammatory processes, during the prenatal and neonatal periods may be involved in the development of ASD. In addition, a number of these studies suggested these relationships may differ based on the presence or absence of co-occurring ID (Additional file 1: Table S2).

### Other endogenous biomarkers

*Thyroid hormones:* Thyroid hormones play a critical role in brain development in regulating neurogenesis, dendrite proliferation, synaptogenesis, and myelination [57–59]. Furthermore, hypothyroidism early in life can lead to intellectual disability, and maternal thyroid conditions have been linked with ASD in prior work [60–64]. In order to address the role of thyroid hormones in ASD, thyroid stimulating hormone (TSH) was examined in maternal samples in phase 1, and neonatal bloodspot TSH in phases 1 and 2 [33, 34]. In these studies we observed inverse relationships between ASD and maternal TSH, as well as early onset ASD specifically [34]. Relationships between ASD and neonatal TSH were not significant, though there was a suggestive inverse relationship with ASD with developmental regression in the phase 2 sample [33].

*Vitamin D:* Vitamin D is a steroid molecule whose primary source is sunlight. Neonatal levels depend on maternal levels, and deficiency has been linked with a range of maternal and child health outcomes [65], including neurodevelopmental outcomes such as cognitive function, language and motor development [66, 67]. Vitamin D plays a well-documented role in immune functioning, including the ability to influence growth and differentiation of innate and acquired immune cells, influence cytokine production, act as an anti-inflammatory agent, and regulate placental inflammation during pregnancy [68, 69]. Vitamin D also plays a role in protecting against oxidative stress, and can act as a neurosteroid in neurodevelopment [70], providing a basis for potential associations with ASD. Thus, for both maternal serum and neonatal vitamin D levels, we hypothesized potential protective effects with higher 25(OH)D levels, or increased risk with vitamin D deficiency [65]. However, primary analyses considering vitamin D deficiency (defined as < 50 nmol/L total 25(OH)D; 14% of newborns and 9% of mothers), insufficiency (defined as 50–74 nmol/L; 26% newborns and 26% of mothers), or according to continuous 25(OH)D levels revealed no significant associations overall at either time point [37, 71]. We also did not observe different associations with ID, or ASD with or without comorbid ID (Additional file 1: Table S2). However, for both maternal and neonatal 25(OH)D levels, there were interactions with child sex and race/ethnicity, such that males and non-Hispanic whites had lower odds of ASD with higher vitamin D levels. In addition, a non-linear, inverted j-shape pattern was observed overall, and among males, between maternal levels and ASD, with the peak around 100 nmol/L 25(OH)D. This pattern was not observed for ID. Of note, 25(OH)D levels were relatively high in EMA (median ~ 85 nmol/L), as may be expected for a study based in relatively lower latitudes (in an area with higher sunlight) [72].

### Environmental toxicants

*EDCs:* Endocrine disrupting chemicals have been hypothesized to influence risk of ASD given evidence for associations with broader adverse neurodevelopmental outcomes. Potential links between these factors are supported by evidence of EDC disruption of thyroid-hormones [73, 74], influence on immune markers, or direct influence on developing neurons [75–78]. Three published analyses have examined classes of organohalogen endocrine disrupting chemicals in association with ASD and ID in EMA [38–40], and one study has examined these associations considering all chemicals simultaneously [79]. Results from individual chemical analyses suggested significant associations with 2 PCB congeners, and a general trend of increases in odds of ASD with levels in the highest quartiles of most PCBs, but no association between ASD and the two organochlorine pesticides (OCP) detected (p,p-D’DE and trans-nonachlor) [40]. No sex differences or differences by ID comorbidity were noted, and the general pattern of associations with PCBs was similar when examining ID as the outcome rather than ASD, though a significant, non-monotonic association was noted between the OCP p’p’-DDE and ID. In contrast, analyses of PBDEs demonstrated null or inverse associations with ASD and ID [39]. When stratified by sex, associations with higher levels of PBDEs were below the null for boys but above for girls, suggesting potential sexual dimorphism (Additional file 1: Table S2). Analyses investigating more mechanistically-defined subgroupings of PCBs and PBDEs (such as dioxin-like or cytokine-P450 inducing PCBs, or highly brominated PBDEs), did not yield differing findings, and thus results did not directly support a specific pathway. For PFASs, associations were generally null, though inverse associations were found for the highest quartile of perfluorooctanoate (PFOA) perfluorooctane sulfonate (PFOS) and ASD [38]. Results were similar for ID and ASD with and without ID, and stratified by sex. Assessing all these chemicals together in single Bayesian model for associations with ASD, no significant associations were found [79]. ORs for the 25 included chemicals were all close to the null and attenuated when compared to analyses based on individual chemicals (Additional file 1: Fig. S1).

*Air pollution:* Air pollution may impact neurodevelopment via inflammatory or oxidative stress pathways, influences on methylation, or via direct effects of specific components on the developing brain [80, 81]. We had two goals in assessing air pollution in EMA: 1) to examine the relationship between maternal cytokine and chemokine levels measured during mid-pregnancy and previous-month (short term) air pollution exposure; and 2) to determine if immune markers measured mid-pregnancy mediate an association between mid-pregnancy air pollutant exposure and child ASD and ID. We found that previous month air pollution exposure and mid-gestational maternal cytokine and chemokine levels were significantly, though weakly, correlated [82]. Some differences by ASD with and without ID, and for ID only, were noted for certain immune marker and pollutant combinations, though overall conclusions were similar across groups. While mid-pregnancy air pollution was not associated with any neurodevelopmental outcome in EMA, we did find that IL-6 remained associated with ASD with ID even after adjusting for air pollution exposure. These results further support a role of maternal immune activation in risk for neurodevelopmental outcomes, and suggest that prenatal air pollution exposure may result in small effects on the immune system with biological relevance.

*Other environmental toxicants*: One study, conducted within the first phase of EMA, examined the association between total mercury, a known neurotoxicant, and ASD [83]. While mean mercury levels were higher in the maternal samples for ASD cases than for GP controls in the unadjusted analysis, differences did not persist after adjustment for covariates, and no case–control differences were noted in neonatal samples. Thus, mercury was not examined in phase 2.

### Genetic factors

**Fig. 3:**
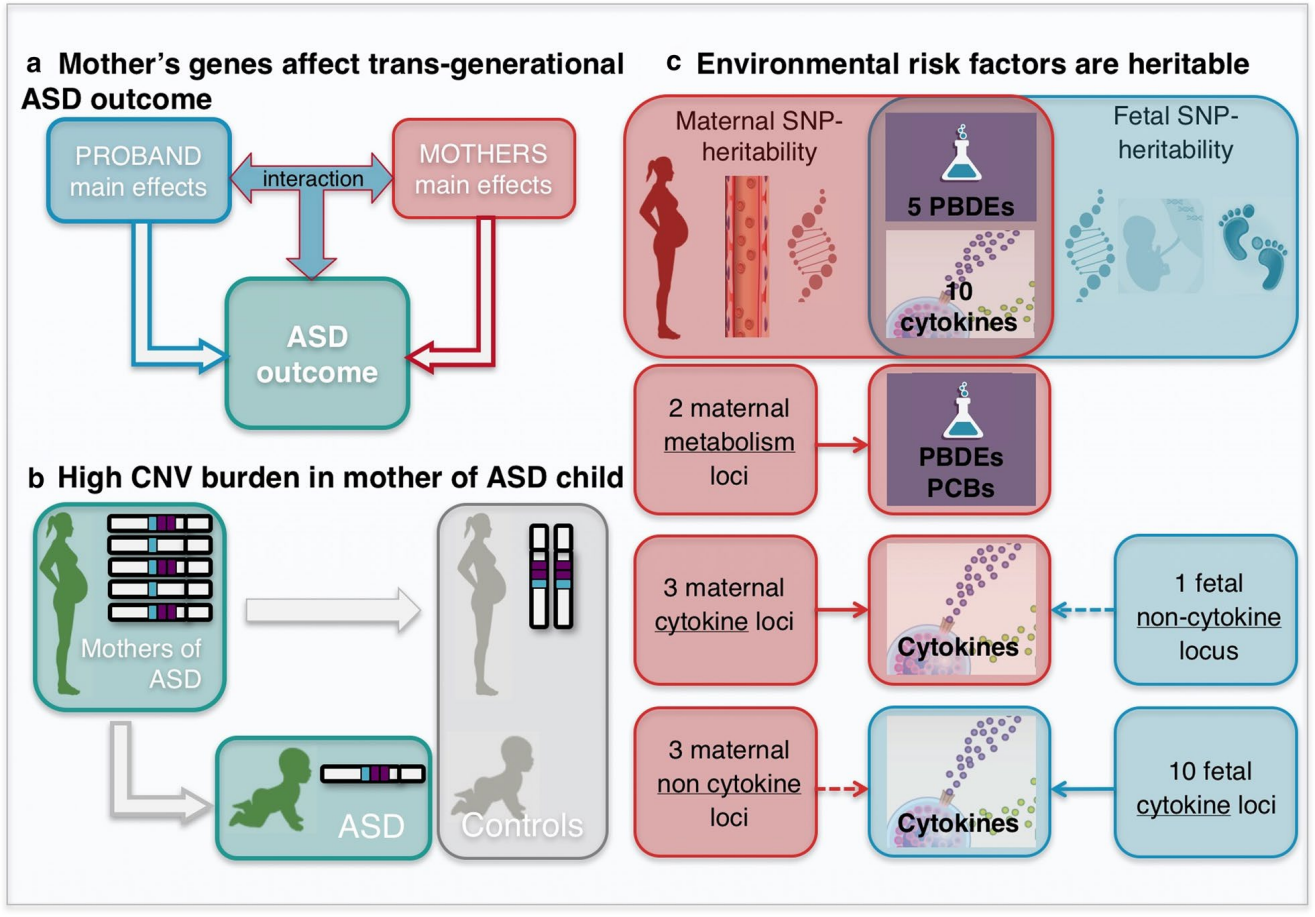
Summary of genetic findings in EMA. CNV, copy number variation; PCB, polychlorinated biphenyl; PBDE, polybrominated diphenyl ether. Further information on relationships summarized in the Figure is as follows: PBDEs associated with maternal and fetal SNP heritability: BB-153, BDE-47, BDE-100, BDE-153, Sum PBDE; cytokines associated with maternal and fetal SNP heritability: IL-4, IL-7, CXCL10, CCL1, CCL3, CCL17, CCL19, CCL22, CCL25, CXCL5; maternal metabolism loci associated with PBDEs and PCBs: *CYP2B6,* between *ADAMTSL1* and *SH3GL2*; maternal cytokine loci associated with maternal cytokines: *THRB/RARB, IL2R, near SMAD1*; fetal non-cytokine loci associated with maternal cytokines: *ADCYAP1*; maternal non-cytokine loci associated with neonatal cytokines: *CYP3A4*, *MCTP2*, near *EFNA5*; fetal cytokine loci associated with neonatal cytokines: *PLCL2, CCL23, CCL15, CXCL9/10/11, CXCL6, CCL24, ACKR4, ACKR2, FBXO25/TDRP, lincRNA*

Genetic factors are known to play a strong role in ASD, with heritability estimates ranging from 50–95%, and evidence of contributions of both rare and common genetic variants [84–86]. Genetic studies in EMA have investigated several hypotheses. First, we applied a novel approach considering maternal genetic effects both alone and in combination with offspring genetic effects. We identified suggestive transgenerational interactions that cannot be explained by maternal or child main effects only [41]; Fig. 3a). We also examined maternal autosomal burden of large, rare copy number variants (CNVs) for transgenerational effects contributing to ASD in children, addressing the hypothesis of a maternal-specific effect (during gestation, rather than interaction between maternal and offspring genotype). We observed that the mothers of children with ASD showed a higher burden of deletions and duplications (> 1 kb) compared with (1) control mothers of unaffected children, (2) both the ASD or control children’s CNV burden, and (3) the enrichment observed in females in the general population [45]; Fig. 3b). Thus, a maternal-specific genetic contribution of excess CNVs in proband was demonstrated.

Second, in order to ultimately assess potential G × E effects, we first applied SNP-based heritability and genome-wide association studies to several E biomarkers. Our biomarker results (unrelated to ASD risk) suggested that a number of environmental prenatal and postnatal biomarkers were highly heritable and/or strongly associated with specific loci [42–44, 49] (Fig. 3c). Results suggested that maternal PBDEs (5/7), maternal (2/22) and neonatal (8/42) cytokines/chemokines and vitamin D circulating during pregnancy and/or at birth are not solely determined by external stimuli (e.g. pollution or infections), but show significant contribution of specific maternal genetic loci relevant for xenobiotic and lipid metabolism [42], immune function [43], and vitamin D metabolism [44], respectively.

Third, we examined the relationships between biomarkers and genetic markers across mothers and neonates, independent of ASD outcome. We found evidence that maternal genetics was associated with neonatal immune biomarkers (3/42), and that fetal genetics had a strong association with mid-gestational maternal immune biomarkers (2/22) [43]. In most cases, we observed independent genetic contribution from the maternal non-transmitted genome and fetal non-inherited genomes and/or a different set of regulatory loci independently associated with maternal or fetal genetic influences. An example is the maternal soluble IL-2 receptor (sIL2-Ra) regulated by maternal genetics through the cytokine-encoding locus (*IL2RA*) and by fetal genetics through a non-cytokine encoding locus (*ADCYAP1*). The nearby *ADCYAP1* gene exerts a transcriptional activating role not previously related to immune function [43]. Additionally, suggestive cross-associated loci influencing maternal and neonatal vitamin D were observed near immune genes. Relevant to pregnancy outcomes, we observed two distinct suggestive interactions between a maternal genetic variant, neonatal vitamin D and ID outcome, and between a second independent maternal genetic variant, neonatal IL-8 and ASD outcome [43, 44].

When we assessed whether the genetic factors controlling the measured biomarkers were associated with ASD outcome, we found none that were statistically convincing (although our power for case–control status was low due to the sample size) [42–44, 49]. However, these studies help to elucidate how genetics strongly influences maternal physiology during gestation with intriguing potential consequences for consideration of the environmental response and its complex interplay with genetics.

## Discussion

The EMA study has reported results for biological markers in association with ASD in over 20 publications to date. By capitalizing on archived biospecimens and widely-available diagnostic outcome data in the state of California, the EMA design enabled a number of “study firsts,” providing novelty in prospective biomarker assessments in ASD research. EMA has allowed researchers to address questions that had previously relied on exposure as estimated through retrospective reporting, biosamples collected after suspected critical windows, and/or had been conducted in small samples. Across work from the EMA study, results support the indication that the prenatal period is one of particular relevance and importance to ASD etiology. EMA has also facilitated comparisons between ASD and ID, specifically suggesting differences in certain immune marker relationships by ASD with and without co-occurring ID, and overall similarity between ASD and ID for associations with EDCs. Several EMA studies have also addressed sex differences in ASD, with differences suggested for vitamin D and PBDEs in particular. Below we further discuss key contributions from the EMA study, as well as future directions for EMA and related work in the field.

Some of the strongest findings from EMA point to the involvement of maternal and newborn immune dysregulation in ASD. Immunologic profiles of mothers of children with ASD, specifically ASD with ID, were marked by elevated levels of several inflammatory cytokines/chemokines that would normally be downregulated during mid-gestation. Neonates with ASD with co-occurring ID were also more likely than ID-only and GP groups to have elevated levels of some inflammatory cytokines/chemokines indicative of heightened immune activation. The findings in EMA demonstrating an association with immune markers and developmental outcome are consistent with a large and growing literature pointing to a role of prenatal and early life immune activation in ASD [87–90]. A primary function of cytokines is to act as growth factors, prime the immune system, and modulate immune activation in response to infection. However, a growing literature supports their key role in mediating signals between the immune and nervous systems, influencing neuronal response and subsequent behaviors as well as brain growth early in development. These maternal and neonatal immune findings in EMA point to promising avenues for understanding the biological mechanisms underlying specific sub-phenotypes of ASD, possible therapeutic targets, and/or early diagnostic biomarkers.

In addition to roles in brain development, cytokines also have been shown to alter neurite outgrowth and synaptic connectivity in cultured rodent neurons [91]. Translational extensions of this work exposing human neuronal cell lines to the 5 cytokines elevated in the EMA ASD with ID group have found that this inflammatory cytokine mix induced apoptosis, altered cytokine receptor expression, and promoted apoptosis [89]. These results indicated that concentration-dependent and interactive cytokine effects from clinically relevant mixtures were shown for the first time to affect developing human neurons, and specifically, to affect endpoints with relevance to ASD [89]. Of future interest is the concerted effect of environmental exposures, such as EDCs, and inflammatory molecules on neuron development.

Only a few other studies have examined prenatal levels of the EDC chemical classes examined in EMA [92–95], some with similar findings to ours, and others with differences. Overall, in our study we found evidence for increased risk of ASD with higher levels of several PCBs, suggestions of sexually dimorphic effects with PBDEs, and overall null findings for OC pesticides and PFASs. We also observed similarity in risk patterns with these EDCs for ID, as well as ASD with and without comorbid ID. Recent work in the Maternal-Infant Research on Environmental Chemicals (MIREC) study in Canada supported EMA findings for PCBs in association with quantitatively-assessed ASD traits according to the Social Responsiveness Scale [96]. Another analysis in the Finnish Prenatal Study of Autism reported no associations between prenatal exposure to PCBs, but did suggest an association with the organochlorine insecticide p’p-DDE [93]. In EMA, p’p-DDE was not associated with ASD, but was associated with ID in a non-monotonic fashion. Mixture analyses examining these classes of EDCs together in our dataset, which have not yet been replicated in other studies, did not reveal any significant associations. The discrepancies in findings according to these different modeling strategies highlight the need for additional work in this area, and suggest either chance findings due to failure to account for correlation across chemicals in the individual chemical class analyses (though findings in those studies did persist in co-exposure models adjusting for one other ‘representative’ chemical from one other class), or perhaps challenges in identifying modest associations in models that necessarily provide conservative estimates when a large number of exposures are examined in a single framework. Because individuals are exposed to hundreds of chemicals, advancement of our understanding of developmental consequences of such exposure may necessitate further assessment of these combined effects.

EMA has also contributed evidence to our understanding of endogenous factors that possibly influence ASD development. Our findings with respect to maternal TSH in mid-pregnancy are generally consistent with literature linking ASD to disrupted thyroid regulation and thyroid conditions in mothers before and during pregnancy [63, 64, 97–99]. However, in the few studies that have directly measured thyroid hormone levels during the prenatal and neonatal periods, the results for associations with neonatal levels are neither consistent nor supportive of strong overall associations [100–103]. Continued work in this area, including studies of environmental and therapeutic influences on the maternal thyroid axis, could provide important insights.

Vitamin D deficiency has been hypothesized as a risk factor for ASD, but early studies were small and often based on estimated exposure (rather than measured levels), or on levels measured outside of relevant developmental windows [104–108]. While results for associations with vitamin D have not been uniform in the broader literature, there is somewhat greater consistency for inverse associations from lower sunlight regions [109]. A recent meta-analysis on neurodevelopmental effects of prenatal vitamin D [110] suggested a protective association (pooled odds ratio of 0.42, 95% CI 0.25, 0.71, for highest vs. lowest prenatal vitamin D). However, only five studies were included, and not all had clinical ASD as the outcome. A few subsequent studies, two of which are also from high sunlight regions, have findings similar to ours, with no overall association between vitamin D deficiency or levels and ASD, but suggestions of a relationship in some sub-groups (such as whites only) [111–113]. Associations with vitamin D should be explored further, as it represents a risk factor with the potential for modification.

Genome-wide genetic data for mothers and their children allowed for several unique mother–child analyses in EMA such as detecting transgenerational effects, GxE interactions, and cross-genetic influence*.* These studies strengthen growing evidence that common variation in particular genes, including SNPs in or near autism-related genes, and maternal burden of CNVs may increase risk of ASD. Our findings at some loci were replicated or are consistent with other work [114]. These include findings in non-pregnant individuals, including relationships between vitamin D and the *GC* gene [104]; *HTR7* variation [104]; and *ACKR1*; as well as relationships between chemokines and *ACKR2* and *ACKR4* [103], and IL-2 and the gene *IL2RA* [103]. However, results for additional maternal and fetal loci regulating neonatal immune biomarkers and vitamin D do not overlap with those from some large studies in European adults [115, 116]. This could be due to unique signals detected in neonates, before environmental variation is large, and in mothers indicating distinct pregnancy biology, or could be due to inclusion of substantial Hispanic and Asian representation in our study but few other GWAS. Finally, when analyzing the cross-genetic contribution to immune biomarkers and vitamin D, the significant and suggestive associated loci mapped to genes that were not already associated with the analyzed immune biomarkers or vitamin D. These results may suggest that the fetal influence on maternal physiology occurs via the placenta (which shares fetal genetics) and distinct regulatory or indirect mechanisms such as signaling at the placental interface and through a different set of genes than currently implicated in inflammation or beyond the classic regulators of the vitamin D pathway.

Our G × E approach has also provided novel, early insights into both maternal and fetal genetic determinants of maternal and neonatal immune regulation, as well as the metabolism of environmental chemicals and vitamin D, separate from pregnancy outcomes. Our findings suggest that studies focusing on risk factors for neurodevelopmental disorders should investigate G × E and cross-genetic interaction involved in early risk for diseases.

### Challenges, limitations, and special considerations in the EMA study

Despite the unique strengths and contributions of the EMA study, certain limitations and considerations for placing findings from this work into the larger context of our understanding of ASD should be noted. The age at ascertainment of Phase 1 cases was on average younger than that for Phase 2, presenting the possibility that Phase 1 cases could represent greater severity. EMA relied on existing diagnostic records from regional centers of the DDS; while these records were reviewed by expert clinicians for consistency with ASD diagnosis, that determination was reliant on available data. Available information from a study that examined the completeness of DDS diagnoses suggests that DDS captures approximately 80% of ASD cases in the state, with milder cases more likely to be missed [117, 118]. We cannot rule out potential misclassification in controls, though any such misclassification would not be expected to influence findings here given the low prevalence of ASD in the general population. Likewise, information on comorbid ID status was reliant on DDS records, and it is possible that some ID cases, or comorbid ID status, may have gone undiagnosed. We do not have data to quantify the extent to which this may occur. We have not been able to examine associations with other comorbidities highly prevalent in ASD, such as anxiety and ADHD, as may be useful to address questions related to associations with other phenotypic subgroups and comorbidites. We had a relatively small number of female ASD cases, limiting power to address sex differences in some analyses. We also lacked in-depth clinical data across the child’s lifespan, preventing the study of developmental trajectories. Certain subgroup analyses (e.g., by sex) may have had more limited statistical power and require replication in larger samples. Many factors examined in phase 1 were not examined in phase 2 participants, precluding ability to pool data across phases. Prenatal confounder information was limited to birth certificate and prenatal screening data, and information on some potential confounders and modifiers, such as parental psychiatric history, or maternal diet, was not available. The EMA design did not include any direct contact with the participants; thus, additional research measures (such as parent-report questionnaires) that may be conducted in ASD case–control studies are not present in our database. These have been the trade-offs enabling a large population-based study with diagnostic information and biospecimens collected during critical developmental windows in mother–child pairs.

As a genetic study, the EMA design and dataset had several relatively unusual features. Though we were able to utilize mother–child pairs to assess some family-based analyses, we did not have paternal genetic contribution to maximize the understanding of which effects might be specific to pregnancy. On the other hand, the availability of unrelated controls allowed for exploration of G × E effects that are challenging in family-based designs. Although the genetic heterogeneity of the dataset created the risk of population stratification, we were able to adjust the analyses for genetic ancestry using the principal components derived from genome-wide maternal and fetal SNPs to account for population stratification, facilitating increased generalizability to the broader US population. The study contributed novel information regarding the relationship between genetic variation and biomarker levels, supporting strong patterns of genetic influence in such levels.

Additional considerations for EMA’s environmental exposure findings, and for placing this study’s results within the larger context of related work, include its geographic location and setting. Our overall null findings for certain factors may have been related to reduced exposure variability; specifically, vitamin D deficiency was low, while air pollution exposure was relatively high, for the Southern California counties included in EMA. EMA’s decision to match cases and controls on month and year of birth (to address concerns related to seasonality in infections, given a key focus of the study was on the immune system’s role in ASD) may also impact exposures that are seasonally associated and reduce ability to detect such associations. For exposures with decreasing (e.g., PBDEs, OC pesticides) or increasing (e.g., PFASs) use, the time period of EMA births (early 2000′s) must also be considered, as varying exposure levels may impact the ability to detect associations, and their observed effect sizes.

Finally, in placing EMA environmental findings in context, combined effects of exposures must also be considered. Environmental exposures vary across populations; these include not only air pollution levels and EDCs, but also other environmental factors not measured here but likely to either influence neurodevelopmentally-relevant pathways directly, or interact with these exposures, such as maternal diet. Such variation means that even in the face of true causal associations, results across studies of environmental risk factors may not be consistent. Future replication of findings should consider such population-level variability and seek to address exposures from a comprehensive standpoint. In compiling a dataset with over 70 biomarkers, many of which are measured in both maternal and child samples, as well as genotypes, the EMA study has been an important step in ASD research towards this overarching aim of considering multiple exposures. Future studies in the -omics and consortium era are likely to build on these findings.

### Future directions and next steps for EMA

The next phases of the EMA study focus on tying together the above exposures to consider pathways and mechanisms, and additional extensions of our combined exposures work. Animal model evidence, and some emerging human studies, suggest that both thyroid hormones and vitamin D may lie along the hypothesized pathway between the environmental chemicals considered in EMA and neurodevelopmental outcomes such as ASD and ID. Emerging work has demonstrated air pollution levels may lead to lower vitamin D levels [119]. Though evidence to date has not provided strong and consistent support for direct associations between thyroid hormones and ASD, thyroid hormones are known to play an important role in neurodevelopment, and links between thyroid hormone levels and EDC exposure have been demonstrated [120]. Ongoing analyses in EMA will examine the potential interaction or mediating effects of vitamin D, as well as TSH, on the associations between chemical exposures and ASD. To our knowledge, EMA will be the first study to examine such pathways in pregnancy for certain classes of chemicals.

In addressing genetic contributions, EMA will also be able to apply our novel G × E and maternal–fetal genetics approaches to additional pre- and post-natal risk factors, including maternal antibodies to fetal brain, to potentially identify novel genetic involvement. In addition to further G × E considerations, EMA has the capacity to examine novel E × E interactions, including not just extensions of existing EDC mixture analyses that may incorporate air pollutant exposures, but also similar strategies to address combined effects of biomarkers available and previously examined only as single exposures (such as, for example, cytokines).

## Conclusions

The EMA study capitalized on existing resources to address what had at one time seemed to be unaddressable questions; that is, well-powered analyses based on biomarker levels from prospectively collected biospecimens from critical periods during neurodevelopment for a rare outcome. Its success highlights the value and utility of resources like the California Biobank Program for archived biosamples and outcome registries like DDS for research purposes. Key findings from EMA include supporting the role of prenatal immune markers in ASD; suggesting potential relationships with levels of certain environmental chemicals; and providing evidence for genetic involvement in regulating or influencing levels of immune markers, endocrine disrupting chemicals, and vitamin D during pregnancy and in the neonatal period. The EMA design has created rich opportunity for analyses of multiple exposures, intermediate biomarkers, and outcomes, and can serve as a model for other studies.

## Supplementary Information

**Additional file 1:** Appendix, Additional Data Tables 1 and 2, and Additional Data Figure 1.

## Data Availability

The datasets used and/or analyzed as part of EMA studies are available from the authors with permission from the California Committee for the Protection of Human Subjects and the California Biobank Program. Genetic data from prenatal and neonatal specimens are not publicly available.

## Abbreviations

ASD: Autism spectrum disorder
CNVs: Copy number variants
DDS: Department of Developmental Services
EDCs: Endocrine disrupting chemicals
EMA: Early Markers for Autism
G × E: Gene by environment
GDSP: Genetic Disease Screening Program
GP: General population
GWAS: Genome-wide association study
ID: Intellectual disability
PBDEs: Polybrominated diphenyl ethers
PCBs: Polychlorinated biphenyls
PM: Particulate matter
RCs: Regional centers
TSH: Thyroid stimulating hormone
