# Supplementary material for: A profile and review of findings from the Early Markers for Autism study: unique contributions from a population-based case–control study in California
Source: Mol Autism. 2021 Mar 18;12:24. doi: 10.1186/s13229-021-00429-7 (PMC7977191; doi:10.1186/s13229-021-00429-7)
Supplement: Supplementary file 1 — Additional file 1: Appendix, Additional Data Tables 1 and 2, and Additional Data Figure 1. [file 13229_2021_429_MOESM1_ESM.docx]

**Additional Data Files for Online Supplement**

Appendix: List of Immune Markers measured in EMA phase 2

Phase 2 markers measured in maternal serum:

Granulocyte macrophage colony-stimulating factor (GM-CSF), IFNγ, IL-1α, IL-1β, IL-2, IL-4, IL-6, IL-7, IL-8, IL-10, IL-12p40, IL-12p70, IL-13, IL-17, IFNγ-induced protein 10 (IP-10), monocyte chemotactic protein-1 (MCP-1), macrophage inflammatory protein-1α (MIP-1α), MIP-1β, tumor necrosis factor-α (TNF-α), eotaxin, soluble IL-2 receptor-α (sIL-2Rα) and IL-1 receptor antagonist (IL-1Rα).

Phase 2 markers measured in neonatal bloodspots:

6CKINE, BCA-1, CTACK, ENA-78, eotaxin-1, eotaxin-2, eotaxin-3, fractalkine, GCP-2, GM-CSF, GRO-A, GRO-B, I-309, IFN-γ, IL-1B, IL-2, IL-4, IL-6, IL-8, IL-10, IL-16, IP-10, I-TAC, MCP-1, MCP-2, MCP-4, MDC, MIF, MIG, MIP-1A, MIP-1D, MIP-3A, MIP-3B, MPIF-1, SCYB16, SDF-1A-B, TARC, TECK, TNF-a, IL-12p70, IL-13

Further details on these markers can be found in Heuer et al, 2019. doi: 10.1016/j.biopsych.2019.04.037.

**Additional Data Table 1.** Comparison of births in study counties to those with a linked prenatal screening specimen and those sampled for the EMA phase 2, by initial and final case status.

|  | Birth Records | | | PNS Specimen-Linked Records | | | EMA Case/Control Final Sample | | | | |
| --- | --- | --- | --- | --- | --- | --- | --- | --- | --- | --- | --- |
|  | DDS ASD | DDS ID | Controls | DDS ASD | DDS ID | Controls | DDS ASD | Final ASD | DDS ID | Final ID | Controls |
| N | 1,094 | 894 | 317,478 | 485 | 428 | 147,416 | 439 | 566 | 374 | 192 | 439 |
|  | % | | | | | | | | | | |
| Sex |  |  |  |  |  |  |  |  |  |  |  |
| Male | 84.1 | 69.0 | 50.9 | 83.3 | 68.0 | 50.7 | 82.5 | 82.2 | 67.4 | 57.3 | 82.5 |
| Female | 15.9 | 31.0 | 49.1 | 16.7 | 32.0 | 49.3 | 17.5 | 17.8 | 32.6 | 42.7 | 17.5 |
| Maternal Race |  |  |  |  |  |  |  |  |  |  |  |
| White Non-HSP | 42.7 | 21.4 | 35.5 | 38.6 | 20.8 | 33.4 | 37.4 | 34.8 | 21.4 | 18.8 | 33.5 |
| Hispanic | 33.0 | 59.6 | 47.2 | 35.5 | 59.6 | 49.8 | 37.6 | 39.9 | 60.7 | 67.7 | 46.0 |
| Black | 4.4 | 5.9 | 3.4 | 2.7 | 5.1 | 2.4 | 3.0 | 2.8 | 5.9 | 6.3 | 2.7 |
| Asian | 16.7 | 10.5 | 11.1 | 19.6 | 11.7 | 11.9 | 18.5 | 18.4 | 9.6 | 6.3 | 13.9 |
| Other | 3.2 | 2.6 | 2.7 | 3.7 | 2.8 | 2.6 | 3.6 | 4.1 | 2.4 | 1.0 | 3.9 |
| County |  |  |  |  |  |  |  |  |  |  |  |
| Imperial | 1.7 | 9.2 | 2.8 | 1.9 | 7.7 | 2.4 | 2.1 | 3.0 | 8.8 | 8.9 | 3.4 |
| Orange | 44.9 | 30.9 | 49.5 | 46.6 | 37.1 | 54.6 | 41.5 | 39.6 | 28.3 | 30.2 | 53.1 |
| San Diego | 53.4 | 60.0 | 47.7 | 51.5 | 55.1 | 43.0 | 56.5 | 57.4 | 62.8 | 60.9 | 43.5 |
| Maternal Age |  |  |  |  |  |  |  |  |  |  |  |
| <20 | 3.4 | 11.2 | 8.3 | 3.1 | 7.9 | 7.7 | 3.4 | 3.0 | 8.3 | 14.1 | 5.5 |
| 20-24 | 13.6 | 20.6 | 21.1 | 13.6 | 19.6 | 20.0 | 14.6 | 15.0 | 19.8 | 23.4 | 16.6 |
| 25-29 | 23.6 | 24.2 | 26.2 | 26.0 | 28.5 | 28.1 | 26.0 | 27.0 | 28.9 | 27.1 | 30.5 |
| 30-34 | 30.8 | 25.4 | 26.8 | 37.3 | 28.3 | 31.3 | 37.1 | 36.2 | 27.3 | 22.9 | 34.4 |
| 35+ | 28.6 | 18.7 | 17.6 | 20.0 | 15.7 | 12.9 | 18.9 | 18.7 | 15.8 | 12.5 | 13.0 |
| Maternal Education |  |  |  |  |  |  |  |  |  |  |  |
| <HS | 13.3 | 35.7 | 26.9 | 14.6 | 35.8 | 28.5 | 15.4 | 17.7 | 35.5 | 42.1 | 24.6 |
| HS | 22.6 | 29.1 | 26.6 | 19.5 | 27.2 | 25.6 | 20.2 | 21.9 | 27.6 | 26.3 | 27.6 |
| College | 44.6 | 27.4 | 35.1 | 45.7 | 28.2 | 34.5 | 43.9 | 41.8 | 28.1 | 25.8 | 34.0 |
| Postgrad | 19.5 | 7.7 | 11.4 | 20.2 | 8.8 | 11.4 | 20.5 | 18.6 | 8.7 | 5.8 | 13.8 |
| Public Insurance |  |  |  |  |  |  |  |  |  |  |  |
| Yes | 25.9 | 51.8 | 40.0 | 25.8 | 54.0 | 40.3 | 27.6 | 31.1 | 56.4 | 62.0 | 36.1 |
| No | 74.1 | 48.2 | 60.0 | 74.2 | 46.0 | 59.7 | 72.4 | 68.9 | 43.6 | 38.0 | 63.9 |
| Parity |  |  |  |  |  |  |  |  |  |  |  |
| 0 | 45.8 | 38.5 | 40.1 | 47.6 | 36.9 | 41.3 | 48.1 | 46.6 | 36.9 | 34.9 | 38.7 |
| 1 | 36.7 | 35.6 | 32.8 | 38.1 | 34.8 | 33.5 | 37.8 | 38.5 | 33.2 | 31.8 | 31.4 |
| 2+ | 17.5 | 26.0 | 27.0 | 14.2 | 28.3 | 25.2 | 14.1 | 14.8 | 29.9 | 33.3 | 29.8 |

**Additional Data Table 2. Summary of secondary findings across EMA studies: Associations with ASD with and without comorbid ID and sex-specific results**

| Category | Factor | ASD with and without ID vs GP | Sex-specific  *(ASD vs GP stratified by sex)* |
| --- | --- | --- | --- |
| Immune-related factors | Maternal cytokines/chemokines  (Jones et al, 2017) | Mothers of children with ASD+ID had elevated mid-gestational levels of numerous cytokines and chemokines, such as GM-CSF, interferon-γ, IL-1α, and IL-6, compared with mothers of children with either ASD-noID or DD or GP controls. Mothers of children with either ASD-noID or with DD had significantly lower levels of the chemokines IL-8 and MCP-1 compared with mothers of GP controls. | Not examined |
|  | Neonatal cytokines/chemokines  (Heuer et al, 2019) | IL-8 associated with both ASD+ID and ASD-noID vs. GP. Higher levels of IL-12p70, eotaxin-1, and GCP-2 noted in ASD-noID vs. GP. IL-4 associated with ASD+ID. | Not examined |
|  | CRP | Not examined | Not examined |
|  | Autoantibodies to fetal brain protein | Not examined | Not examined |
|  | Immunoglobulins | Not examined | Not examined |
| Additional endogenous factors | BDNF | Not examined | Not examined |
|  | Maternal TSH | Not examined | Not examined |
|  | Neonatal TSH  (Ames et al, 2020) | No major differences by ASD with and without ID. | Not examined |
|  | Maternal Vitamin D  (Windham et al, 2020) | No major differences by ASD with and without ID. | 25(OH)D associated with lower odds of ASD in males and higher odds in females. |
|  | Neonatal Vitamin D  (Windham et al, 2019) | No major differences by ASD with and without ID. | 25(OH)D associated with lower odds of ASD in males and higher odds of ASD in females. |
| Environmental chemicals | Metals (Mercury) | Not examined | Not examined |
|  | PCBs  (Lyall et al, 2017a) | No major differences by ASD with and without ID. | No clear patterns noted. |
|  | OCPs  (Lyall et al, 2017a) | No major differences by ASD with and without ID. | No clear patterns noted. |
|  | PBDEs  (Lyall et al, 2017b) | No major differences by ASD with and without ID. | Positive associations for girls in all congeners (though not statistically significant) and inverse associations for boys. |
|  | PFASs  (Lyall et al, 2019) | No major differences by ASD with and without ID. | No differences noted. |
|  | Mixtures  (Hamra et al, 2019) | No major differences by ASD with and without ID. | No differences noted. |
|  | Air pollution  (Volk et al, 2020) | Some differences by ASD with and without ID. | Not examined |
| Genetic factors | GWAS, CNVs, candidate genes | Not examined | Not examined |

**Additional Data Figure 1. Comparison of mixture and individual chemical results for associations between EDCs and ASD**

**
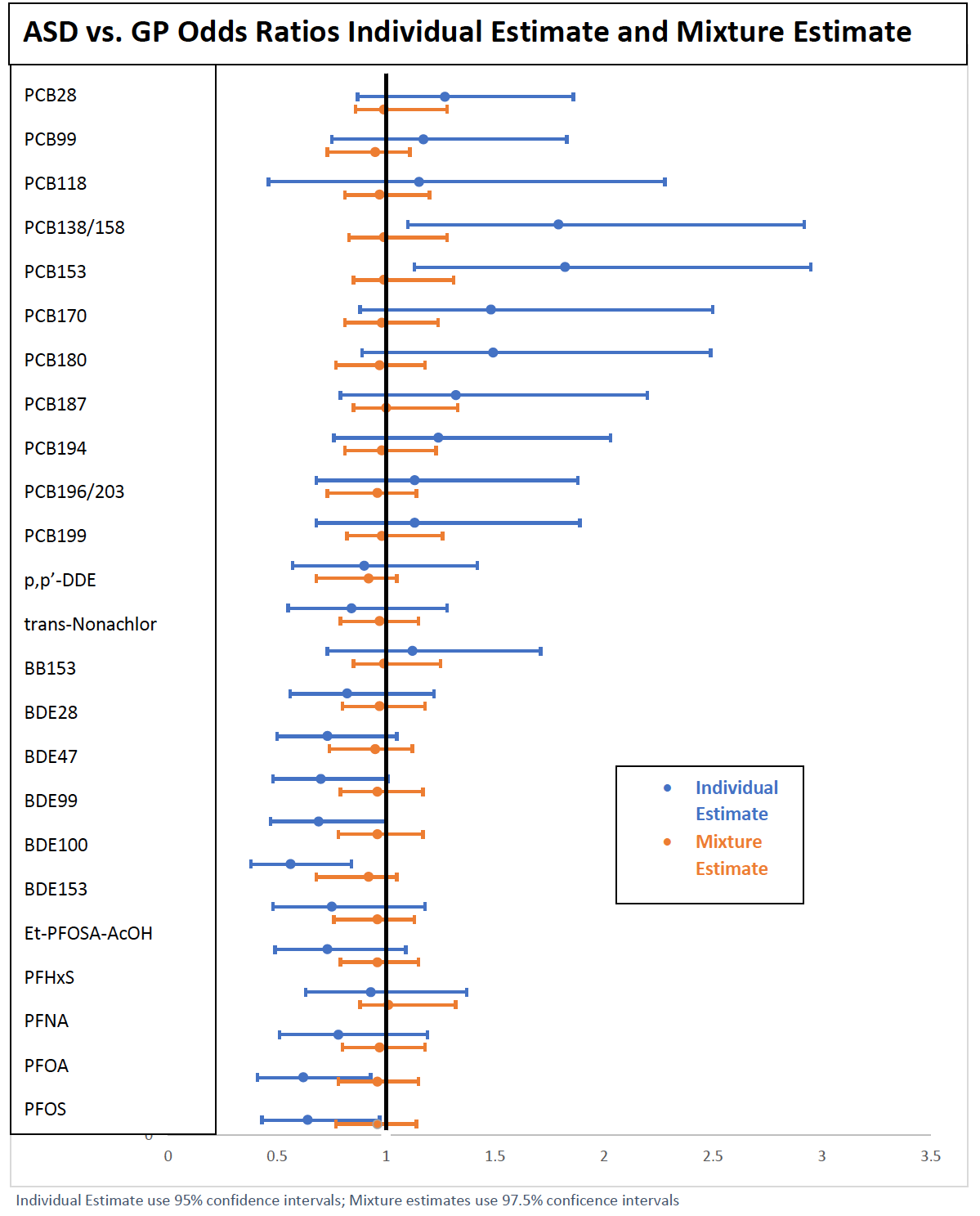
**

Adjusted odds Ratios of the association between highest vs. lowest quartiles of EDC exposures and ASD (relative to GP controls)

Additional Data Figure 1 legend: Forest plot comparing adjusted odds ratios (circles) and their confidence intervals (lines) obtained from studies of individual chemicals (in blue; Lyall et al, 2017a and b; Lyall et al, 2019) and mixture estimates (in orange; Hamra et al, 2019). Analyses of individual chemicals utilized 95% confidence intervals; mixture analyses used 97.5% confidence intervals.
